# Radial nerve injury causes long-lasting forelimb sensory impairment and motor dysfunction in rats

**DOI:** 10.1097/PR9.0000000000000957

**Published:** 2021-09-16

**Authors:** Katherine S. Adcock, Daniel R. Hulsey, Tanya Danaphongse, Zainab Haider, Robert A. Morrison, Michael P. Kilgard, Seth A. Hays

**Affiliations:** a Texas Biomedical Device Center, The University of Texas at Dallas, Richardson, TX, USA; b School of Behavioral and Brain Sciences, The University of Texas at Dallas, Richardson, TX, USA; c Department of Bioengineering, Erik Jonsson School of Engineering and Computer Science, The University of Texas at Dallas, Richardson, TX, USA

**Keywords:** Peripheral nerve injury, Radial, Forelimb, Motor, Sensory

## Abstract

**Introduction:**

Peripheral nerve injury is a common cause of lifelong disability in the United States. Although the etiology varies, most traumatic nerve injuries occur in the upper limb and include damage to the radial nerve. In conjunction with the well-described effects of peripheral damage, nerve injuries are accompanied by changes in the central nervous system. A comprehensive understanding of the functional consequences of nerve injury is necessary to develop new therapeutic interventions.

**Objectives:**

We sought to characterize changes in sensory and motor function and central neurophysiology after radial nerve injury in rats.

**Methods:**

To evaluate somatosensory function in the forelimb, we assessed mechanical withdrawal threshold, spontaneous forelimb use, and cold sensitivity in rats 10 and 16 weeks after radial nerve injury. To evaluate motor function, we assessed performance on a forelimb supination task for up to 16 weeks after nerve injury. Physiological changes in the motor and somatosensory cortex were assessed using intracortical microstimulation and multiunit recordings, respectively.

**Results:**

Our results indicate that radial nerve injury causes long-lasting sensory and motor dysfunction. These behavioral deficits are accompanied by abnormal cortical activity in the somatosensory and motor cortex.

**Conclusion:**

Our results provide a novel characterization of functional deficits that are consistent with the clinical phenotype in patients with radial nerve injury and provide a framework for future studies to evaluate potential interventions.

## Abstract 2

Radial nerve injury in rats produces lasting sensorimotor impairments and cortical dysfunction that is consistent with the clinical phenotype in nerve-injured patients.

## 1. Introduction

Traumatic injury to peripheral nerves often results in significant disability. Nerve injuries commonly decrease overall quality of life because of motor and sensory dysfunction, particularly when pertaining to the hand.^13,22,30^ The radial nerve is one of the most commonly injured of the forelimb nerves and leads to the most debilitating consequences.^14,36,41^ Many patients exhibit a loss of mobility in the supination and wrist extensor muscles. In addition, patients experience loss of sensation to the posterior arm and the dorsal aspect of the hand, which can be accompanied by pain.^18,21^

A number of preclinical studies have characterized motor and sensory dysfunction after nerve injury in animal models, and the resulting deficits are largely consistent with clinical features of traumatic nerve injury.^42^ Although most studies focus on injury of nerves in the hindlimb, some preclinical studies have shifted to evaluating forelimb nerve injuries, which is more reflective of the clinical population of traumatic nerve injuries. Damage to the radial nerve accounts for most traumatic nerve injuries in the forelimb; thus, a comprehensive understanding of the consequences of radial nerve injury is necessary to develop interventions.

Several studies revealed long-lasting changes in the central nervous system in response to nerve injury for both the motor and sensory systems.^7,12,23,27,38,40,43,44^ However, little is known about the nature of these changes in the context of radial nerve injury. We therefore sought to explore central nervous system changes that may accompany radial nerve injury.

In the current study, we sought to develop a model of peripheral nerve injury (PNI) that is representative of what is commonly seen in patients and assessed sensory and motor function after radial nerve injury in a rat model. We observed that radial nerve injuries produce lasting motor and sensory impairments. This behavioral dysfunction accompanies changes in somatosensory and motor cortices. These results provide a novel characterization of functional deficits that are consistent with the clinical phenotype in patients who have radial nerve injury. Furthermore, this work provides a framework for future studies to evaluate potential interventions to restore motor and sensory function after damage to the radial nerve.

## 2. Methods

### 2.1. Subjects

Thirty-six adult female Sprague-Dawley rats were used in this study, each weighing approximately 250 g. Twenty-two animals were injured, and 14 were used as uninjured controls. Nine nerve-injured animals were used for mechanical sensory testing, cold, cylinder, and grip strength. Eight nerve-injured animals were used for the supination assessment task. Eight nerve-injured animals and 10 uninjured controls were used for sensory neurophysiology. Five nerve-injured animals and 4 uninjured controls were used for intracortical microstimulation (ICMS). All animals were housed in a 12:12-hour reversed light–dark cycle and were food deprived during motor training. All protocols were approved by The University of Texas at Dallas Institutional Animal Care and Use Committee.

### 2.2. Peripheral nerve injury

**Figure 1.**
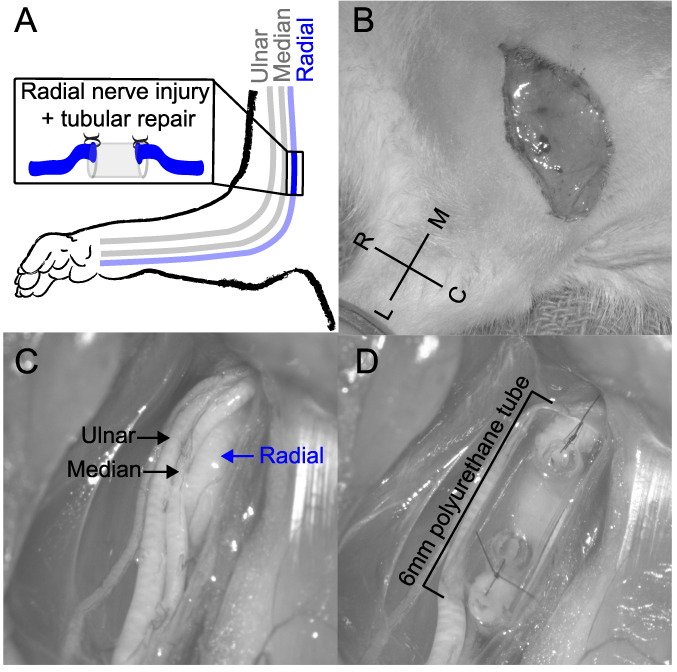
Images of the radial nerve injury procedure. (A) Illustration of the radial nerve injury in the right forelimb of the rat. (B) A small incision on the forelimb proximal from the elbow was made. (C) The radial nerve was carefully isolated, exposed, and completely transected. (D) The proximal and distal stumps of the nerve were sutured 1 mm inside the opposite ends of a 6-mm saline-filled polyurethane tube, resulting in a 4-mm gap between nerve stumps.

Peripheral nerve injuries were performed on the right forelimb. Complete transection of the radial nerve proximal to the elbow followed by tubular repair was performed (Fig. 1). Animals were deeply anesthetized with ketamine hydrochloride (80 mg/kg, intraperitoneally [i.p.]) and xylazine (10 mg/kg, i.p.) and given supplemental doses as needed to maintain areflexia. A small incision on the forelimb proximal from the elbow was made, and the radial nerve was carefully isolated, exposed, and completely transected with microscissors. Immediately after transection, the proximal and distal stumps of the nerve were sutured 1 mm inside the opposite ends of a 6-mm saline-filled polyurethane tube (Micro-Renathane 0.095″ I.D 0.066″ O.D., Braintree Scientific, Inc, Braintree, MA), resulting in a 4-mm gap between nerve stumps. The skin incision was sutured and treated with antibiotic ointment. All animals were given a single injection of sustained-release buprenorphine (1.2 mg/kg, i.p.) and enrofloxacin (7.5 mg/kg, i.p.) immediately after surgery.

### 2.3. Forelimb mechanical sensory test

Mechanical withdraw thresholds were assessed before PNI, 10 weeks after PNI, and 16 weeks after. Testing was performed in an acrylic chamber on a wire mesh floor. Forepaw mechanical sensitivity was tested on the right and left forepaws using a Dynamic Plantar Aesthesiometer (Ugo Basile, Italy), which automatically detects and records latency time and force at the time of paw withdrawal.^6,15,27^ The movable actuator filament (0.5-mm diameter) is positioned below the plantar (ventral) surface of the forepaw, and a linearly increasing force was applied (20-second ramp time, 50-g maximal force). The force at which the paw withdrawal occurred was recorded for analysis. The left paw and right paw were alternately tested, with a minimum 1-minute interval between consecutive tests. The average force over 5 trials was calculated for each paw.

### 2.4. Cold withdraw latency test

Withdrawal latencies of the forelimb in response to a cold stimulus were assessed before PNI, 10 weeks after PNI, and 16 weeks after. Testing was performed in an acrylic chamber on a wire mesh floor. Cold withdrawal latency was tested on the right and left forepaws. An ice probe was made by freezing water in a 0.6-mL tube with a plastic applicator stick frozen into the ice for a handle.^20^ The ice probe was applied to the plantar surface of the forepaw under the mesh floor, and a stopwatch was used to measure the latency to withdraw from the ice probe. The left paw and right paw were alternately tested, with a minimum 1-minute interval between consecutive tests. The average latency over 5 trials was calculated for each paw.

### 2.5. Cylinder forelimb asymmetry test

Spontaneous use of forelimbs during exploratory activity was measured before and after PNI using the cylinder forelimb asymmetry task, similar to previous descriptions.^6,39^ Animals were placed in a clear cylinder (20-cm diameter) and allowed to explore for 3 minutes. Video was recorded from under the cylinder through a clear sheet of acrylic. The total number of both left and right forepaw touches was recorded. An asymmetry index, describing the relative use of the injured forelimb, was calculated as 100 × right forepaw touches ÷ (right forepaw touches + left forepaw touches).

### 2.6. Grip strength testing

A custom-made grip strength meter was used to measure the grip strength of the right and left forepaws independently, similar to previous descriptions.^9^ The rat was positioned over the horizontal bars attached to separate force transducers such that each forepaw grasped a single bar. Rats were held horizontally suspended by their hindquarters and slowly pulled away from the bars until their grip broke. The peak force at which grip was released from the bar was recorded for each paw individually. Five trials were performed at each assessment, and the average of the peak grip forces was recorded.

### 2.7. Supination assessment task

Animals underwent training in the supination task as previously described.^25,28,33^ Training sessions occurred twice a day for 30 minutes each, 5 days a week. The behavioral training apparatus consisted of an acrylic cage with a slot in the front right in which animals will reach out of a slot, grasp, and supinate their forelimb to rotate a spherical manipulandum. The manipulandum was affixed to a rotary encoder that provides turn angle measurements. Success rate was defined as trials in which the turn angle exceeds 60°. Training continued until animals achieved a 75% success rate, averaged across 6 consecutive training sessions. Once this criterion was met, animals underwent surgery in which the radial nerve was injured. After a 10-week recovery period, animals were reassessed on the supination task for 10 sessions with at least 50 trials each session, with this data being used for the post-time point in all analyses. Animals continued training for 6 more weeks (16 weeks after injury).

### 2.8. Primary somatosensory cortex recording and mechanical digit stimulation

Rats underwent primary somatosensory cortex (S1) recordings to evaluate somatosensory responses and cortex organization. Rats were anesthetized with ketamine hydrochloride (75 mg/kg, i.p.) and xylazine (5 mg/kg, i.p.) and mounted into a stereotaxic frame.^5^ Supplemental doses were administered as necessary. A small incision of the cistern magna was made to attenuate cortical swelling. A craniotomy and durotomy exposed left S1, which was covered with silicone oil to prevent drying. The right forepaw was glued in a natural position to a podium with a nearly vertical plane, exposing the glabrous side of the paw and providing access to digits 2 to 5. The recording procedure was performed as previously described.^15^ Electrodes were lowered to approximately 650 µm below the pial surface to record multiunit spiking activity in layer IV of the cortex. At each recording site, individual mechanical tactile stimulation of digits 2 to 5 was delivered 20 times at 2 Hz in a randomly interleaved order using the electromagnetic devices described previously.^15^ The contiguous digit region was mapped completely and was constrained by recording sites with lower lip, D1, thenar, palmar, or hypothenar pad receptive fields or by sites with no discernable receptive fields.

The preferred digit at each recording site was determined by the maximal number of driven spikes in response to individual digit stimulation. Response periods were defined for each stimulation type from an average peristimulus time histogram. Driven spikes for each stimulation type were defined as the driven spike rate (mean response period–mean spontaneous period [1–90 ms]) × response duration (end of response latency − onset latency).

### 2.9. Intracortical microstimulation

Rats underwent ICMS to evaluate left motor cortex organization contralateral to the injured paw, using standard procedures.^11,27,34^ Rats were anesthetized with ketamine hydrochloride (75 mg/kg, i.p.) and xylazine (5 mg/kg, i.p.), with supplementary doses given as needed to maintain anesthesia levels. Doxapram (20 mg/kg, i.p.) and glycopyrrolate (0.5 mg/kg, i.p.) were given to stabilize breathing and heart rate as needed. A small incision of the cistern magna was made to attenuate cortical swelling. A craniotomy and durotomy was performed to expose the left motor cortex. A tungsten electrode (0.1–1 MΩ) was inserted into the brain at a depth of 1.75 mm. Stimulation sites were then chosen at random on a grid with sites set 500 μm apart from each other.

Intracortical microstimulation procedures were conducted with 2 experimenters to ensure blinding to group and electrode location. The first experimenter placed the electrode and recorded data from each site. The second experimenter, blinded to electrode position, delivered stimulations and classified movements. Each stimulation consisted of a 40-ms pulse train of 10 pulses. Stimulation intensity was gradually increased from 20 μA to 250 μA or until a movement was observed. The stimulation intensity at which a movement was first seen was documented as the threshold. If no movement was seen at 250 μA, then that site was recorded as no response. Movements were classified as proximal forelimb, distal forelimb, or nonforelimb. Cortical area was calculated by multiplying the number of sites eliciting a response by the area surrounding a site (0.25 mm^2^).

### 2.10. Statistical analysis

Statistical analysis was performed with MATLAB software. One-way repeated-measures analyses of variance (ANOVAs) were used to analyze mechanical withdrawal thresholds, cold sensitivity, grip strength, and forelimb motor performance over time. Post hoc paired *t* tests were used to determine differences before and after nerve injury. Comparisons were Bonferroni corrected for the number of time points where appropriate. To assess ICMS data and S1 recording data, an unpaired *t* test was used to determine significance between groups. All data are reported as mean ± SEM. An additional 10 animals failed to demonstrate a forelimb motor deficit, defined previously as an average postlesion baseline performance with at least 30% of trials exceeding 60° on the supination task, and were excluded.^33^

## 3. Results

**Figure 2.**
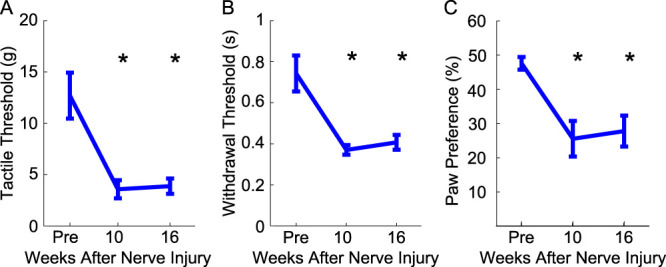
Radial nerve injury impairs sensorimotor forelimb function. (A) Mechanical withdraw threshold was significantly reduced after radial nerve injury, indicating hypersensitivity to mechanical stimulation. (B) Time to withdraw from a cold stimulus was also reduced after radial nerve injury, indicating sensitivity to cold. (C) Radial nerve injury caused an increased reliance on the uninjured paw in the cylinder task. All plots show group averages (N = 9). Error bars indicate SEM. *P < 0.02.

We first sought to evaluate the impact of radial nerve injury on somatosensory function in the forelimb. To do so, we assessed mechanical withdraw threshold, spontaneous forelimb use, and cold sensitivity in rats 10 weeks after transection and gap repair of the radial nerve proximal to the elbow. Radial nerve injury caused long-lasting mechanical hypersensitivity to the ventral surface of the forepaw for up to 16 weeks, as evidenced by a reduction in forelimb mechanical withdraw thresholds (Fig. 2A, one-way repeated-measures ANOVA, F[2,16] = 12.65, *P* = 0.0005, post hoc paired *t* tests, pre vs weeks 10 and 16, *P* < 0.025). Similarly, radial nerve injury resulted in more rapid withdrawal to a cold stimulus, indicative of an increased sensitivity to cold (Fig. 2B, one-way repeated-measures ANOVA, F[2,14] = 11.66, *P* = 0.001, post hoc paired *t* tests, pre vs weeks 10 and 16, *P* < 0.025). Sensorimotor function was also impaired by radial nerve injury. After injury, rats exhibited an increased reliance on the uninjured forelimb during the cylinder test (Fig. 2C, one-way repeated-measures ANOVA, F[2,14] = 14.087, *P* = 0.0004, post hoc paired *t* tests, pre vs weeks 10 and 16, *P* < 0.025). These results are consistent with other models of PNI.^2,45^

**Figure 3.**
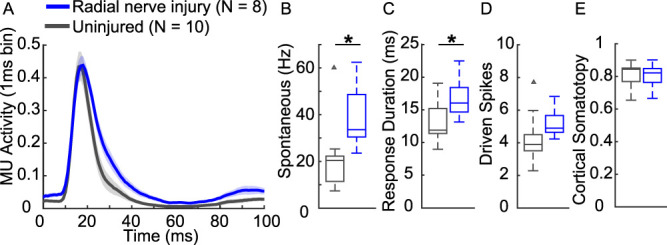
Radial nerve injury causes modest, but significant changes in the forelimb primary somatosensory cortex. (A) Average poststimulus time histogram of preferred digit responses. (B) Radial nerve injury did not influence overall response strength. (C) Radial nerve injury caused higher spontaneous firing rate and (D) longer evoked response duration. (E) However, there are no changes in cortical somatotopy, indicating no apparent large-scale changes in cortical organization. Box plots show the median and interquartile ranges. Triangles indicate outliers (uninjured N = 10; radial nerve injury N = 8). *P < 0.05. MU, multiunit.

A number of pioneering studies reveal lasting changes in the central nervous system in response to nerve injury,^12,23,40,43,44^ but relatively little is known about the nature of these changes in the context of radial nerve injury. Given that radial injury produced chronic deficits in somatosensory behaviors, we sought to examine the effects on somatosensory networks. To do so, we performed multiunit recordings in the primary somatosensory cortex (S1) contralateral to the injured limb in response to mechanical stimulation of the digits (Fig. 3A). Radial nerve injury resulted in a significantly higher spontaneous firing rate compared with recordings in uninjured animals (Fig. 3B, unpaired *t* test, injured: 38.9 ± 5.2 Hz, uninjured: 21.3 ± 4.8 Hz, *P* = 0.025). In addition, radial nerve injury caused a longer evoked response duration (Fig. 3C, unpaired *t* test, injured: 16.74 ± 1.05 ms, uninjured: 12.81 ± 1.04 ms, *P* = 0.01). However, radial nerve injury did not influence overall evoked response strength (Fig. 3D, unpaired *t* test, injured: 5.18 ± 0.307, uninjured: 4.27 ± 0.48, *P* = 0.15). We next evaluated organization of sensory representations in S1 to determine whether radial nerve injury disrupts cortical organization of the ventral surface of the digits. Somatotopy of the digit representations in S1 was determined by calculating the best coefficient of determination for preferred digit response organization along a linear axis, as previously described.^15^ Both injured and uninjured animals were highly organized (Fig. 3E, unpaired *t* test, injured: 0.80 ± 0.02, uninjured: 0.81 ± 0.02, *P* = 0.78).

**Figure 4.**
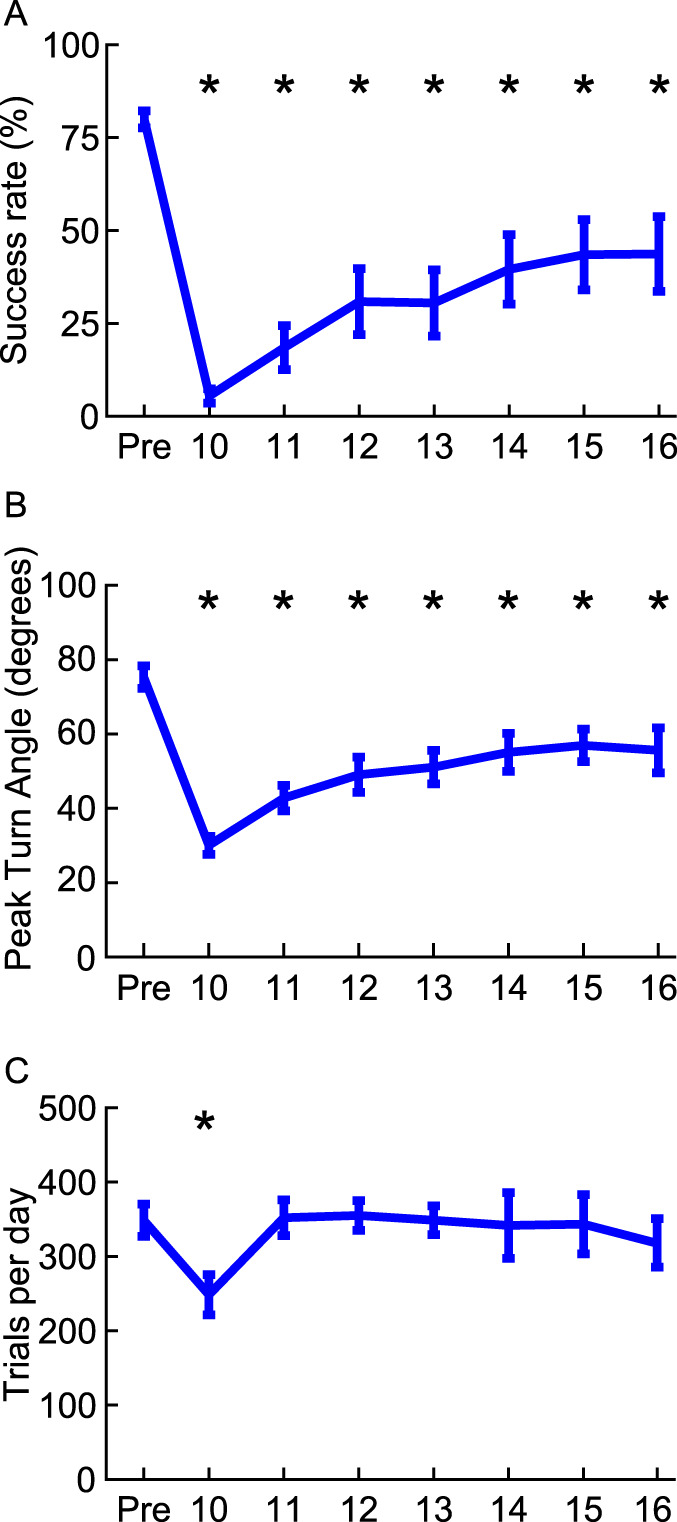
Radial nerve injury impairs skilled forelimb motor function. After radial nerve injury, animals had (A) reduced performance on the supination task and (B) exhibited a reduction in peak turn angle, reflecting a loss of supination range of motion. (C) Injury also caused a transient reduction in the average number of trials the animals performed per day. All plots show group averages (N = 8). Error bars indicate SEM. *P < 0.007.

We next sought to classify the influence of radial nerve injury on skilled forelimb motor function. The radial nerve provides innervation of the supinator and wrist extensors; thus, we assessed performance on task that requires grasping and supination of the forelimb.^25^ Once animals were trained to proficiency on this task they underwent a radial nerve injury. Ten weeks after injury, animals returned to evaluate forelimb function. Radial nerve injury significantly impaired motor performance on the supination task and exhibited a significant reduction in peak turn angle (Fig. 4A, B, one-way repeated-measures ANOVA, success rate: F[7,49] = 19.52, *P* = 3.42e-12, post hoc paired *t* tests, pre vs weeks 10 to 16, *P* < 0.007; peak turn angle: F[7,49] = 23.26, *P* = 1.5007e-13, post hoc paired *t* tests, pre vs weeks 10–16, *P* < 0.007). Radial nerve injury also had a transient effect on the average number of trials the animals performed per day (Fig. 4C, one-way repeated-measures ANOVA, F[7,49] = 3.08, *P* = 0.008, post hoc *t* test, pre vs week 10, *P* = 0.002). In addition, radial nerve injury produced a trend toward forelimb weakness, as measured by reduced grip strength, but this measure failed to reach statistical significance (one-way repeated-measures ANOVA, F[2,10] = 3.52, *P* = 0.06).

**Figure 5.**
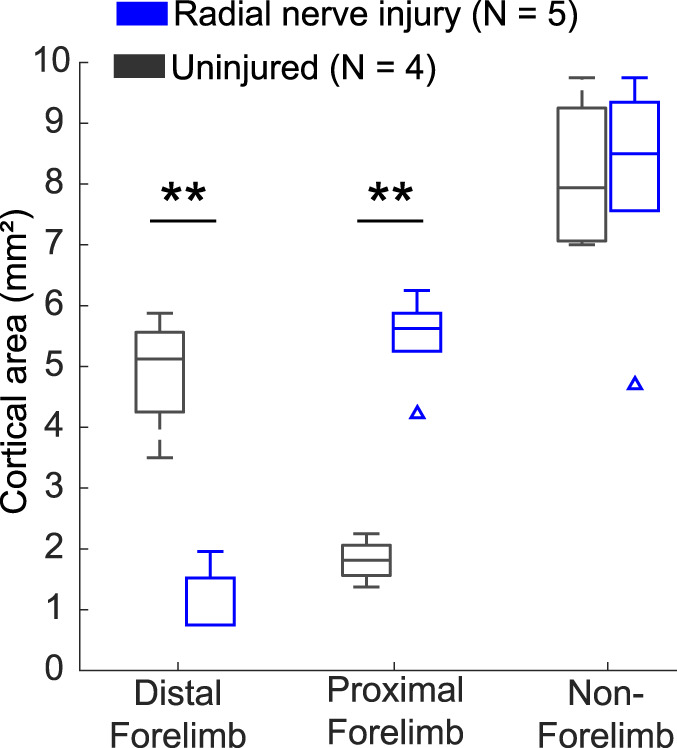
Radial nerve injury causes reorganization of cortical motor networks. Radial nerve injury caused a reduction in cortical area that evokes movements of the distal forelimb and an expansion in cortical area that evokes movements of the proximal forelimb. No difference was observed between groups in cortical area that evokes nonforelimb movements. Box plots show the median and interquartile ranges. Triangles indicate outliers (uninjured N = 4; injured N = 5). **P < 0.001 across groups.

Based on these motor impairments, we next sought to characterize changes in cortical motor networks using ICMS. Radial nerve injury caused a reduction in cortical area that evoked movements of the distal forelimb and an expansion in cortical area that evoked movements of the proximal forelimb (Fig. 5, unpaired *t* test, distal: injured: 1.11 ± 0.24 mm^2^, uninjured: 4.9 ± 0.5 mm^2^, *P* = 1.66E-04; proximal: injured: 5.49 ± 0.33 mm^2^, uninjured: 1.81 ± 0.18 mm^2^, *P* = 4.27E-05). There was no difference between groups in cortical area that evoked other nonforelimb movements (Fig. 5, unpaired *t* test, injured: 8.14 ± 0.87 mm^2^, uninjured: 8.15 ± 0.66 mm^2^, *P* = 0.99). These findings corroborates previous studies reporting cortical changes in response to nerve injury.^7,28,38^

## 4. Discussion

In this study, we sought to develop a rat model of nerve injury that is representative of what is commonly seen in patients with PNI. We report long-lasting forelimb sensory and motor dysfunction after radial nerve injury, which was accompanied by changes to the central nervous system. Together, these findings illustrate that this injury model mirrors common deficits seen in patients with radial nerve injury and provides a framework for development of therapies to target this dysfunction.

Pain is a common complaint that accompanies peripheral nerve damage.^17,18,29^ We find that rats exhibit mechanical hypersensitivity for up to 16 weeks after radial nerve injury. These findings mirror those observed in other nerve injury models in the hindlimb, including the spared nerve injury (SNI) model. Spared nerve injury of the hindlimb is the standard animal model for PNI.^42,45^ In SNI, the common peroneal and tibial nerves of the sciatic branch are injured, causing sensory hypersensitivity for up to 31 weeks in the portion of the hindlimb that is innervated by the intact sural nerve.^2,45^ We sought to extend the SNI model to the forelimb to mimic pain and motor dysfunction that is commonly observed in patients with upper limb nerve injury, specifically radial nerve injury. The current study injured the radial nerve of the forelimb, leaving the median and ulnar nerves intact. Similar to the SNI model, injury to the radial nerve produced long-lasting mechanical hypersensitivity in the ventral side of the forepaw, which is innervated by the intact median and ulnar nerve. We expect that radial nerve injury would produce loss of sensation in the dorsal aspect of the forepaw, which is innervated by the injured radial nerve, but there are no common and well-validated means to test this. Thus, in keeping with our goal of developing a model of forelimb pain, we elected to test the palmar aspect of the paw to test the hyperalgesia of the uninjured median and ulnar nerve. In addition to abnormal mechanical sensation, sensitivity to cold is commonly exhibited by patients with neuropathic pain after nerve damage.^4,16,24,31^ The current study found that radial nerve injury caused long-lasting cold sensitivity, consistent with previous studies.^3,10,45,46^ Recapitulation of this key feature observed in patients provides validity for testing interventions to reduce pain after nerve damage in this model.

Sensory dysfunction after nerve damage is often accompanied with long-lasting changes to the central nervous system.^12,23,40,43,44^ We explored potential central nervous system changes in the primary somatosensory cortex after radial nerve injury. The current study also found increased spontaneous neural activity and lengthened response durations. Although previous studies observed cortical reorganization after injury to one of the forelimb nerves,^15,23,40,43,44^ we observed no significant changes in digit organization after radial nerve injury. This likely arises from the fact that the median and ulnar nerves that provide the primary innervation of these networks are still intact. Therefore, the spared input to these circuits may prevent large scale reorganization or changes in evoked strength. In addition, limitations of the recording technique used to evaluate sensory function provide only a relatively coarse and noncomprehensive assessment of evoked sensory activities.

Chronic loss of motor control and diminishment of strength is common after nerve injury.^8,21,35–37^ The radial nerve provides innervation of muscles involved in rotation of the forearm; thus, patients are often unable to effectively supinate the hand after injury.^18^ We sought to explore whether similar impairment of forelimb rotation could be measured in rats. Animals with radial nerve injury exhibited lasting reductions in forelimb supination that lasted for at least 16 weeks. In addition to control of forelimb rotation, muscles innervated by the radial nerve are responsible for elbow and digit extension. Consistent with this, previous studies report impairments in reach and grasp tasks in rats after various forelimb nerve injuries.^32^ Similarly, injury to the median and ulnar nerves which are primarily responsible for grasping caused reduced motor performance for up to 12 weeks on a task that requires an animal to reach out, grasp, and pull on a handle.^26^ These results are consistent with the chronic motor dysfunction seen in patients.

Previous studies indicate that nerve damage also causes robust motor network reorganization of the forelimb area.^7,28,38^ We explored whether similar reorganization would co-occur with radial nerve injury. In alignment with previous studies, we observed a reduction in cortical area that evoked movements of the distal forelimb and an expansion in cortical area that evoked movements of the proximal forelimb. Elbow flexion, a proximal movement, is mainly controlled by the intact musculocutaneous nerve, whereas the injured radial nerve controls distal movements involved in supination or digit extension. The expansion of movements controlled by intact nerves at the expense of movements on controlled by the damaged nerve, even after reinnervation, is a common finding.^19^ Recent evidence highlights the importance of this cortical reorganization in the restoration of motor function after nerve injury.^27^

In the current study, we observed that radial nerve injury produced robust motor impairments on a forelimb supination task in 8 animals. However, 10 animals failed to demonstrate a forelimb motor deficit after nerve injury. Compensatory action of other muscles may underlie the absence of an impairment in the observed supination task performance. Because radial nerve injury results in an expansion of proximal movements, it is plausible that compensation with the muscles involved in movements may underlie the absence of behavioral deficits. This expansion may be driven by the intact musculocutaneous nerve, reinnervated fibers from the radial nerve, or a combination thereof. More detailed electromyography recordings in future studies may provide a means to delineate muscle function after radial nerve injury and could be valuable in terms of clinical assessment.

A number of limitations of this study merit consideration. The current study characterized motor and sensory function in a novel female rat model of PNI that is representative of what is commonly seen in patients. However, a recent study observed sexual dimorphism in the development of mechanical and cold allodynia after PNI in rats.^1^ Future studies that expand evaluation of radial nerve injury in both sexes will provide a more comprehensive assessment of forelimb dysfunction depicted in this model. In addition, the current study did not include a sham injury group to assess stable performance in the behavioral measures in uninjured animals.

A comprehensive understanding of the functional consequences of nerve injury is necessary to develop new therapeutic interventions. In the current study, we sought to develop a rat forelimb model of radial nerve injury (PNI) that is representative of some aspects of clinical presentation, including loss of motor function and pain. We observed that radial nerve injuries produce lasting motor and sensory impairments. This behavioral dysfunction accompanies changes in somatosensory and motor networks in the brain. Our findings provide a framework for future studies to determine potential interventions that improve impairments after nerve damage, which could improve the quality of life of patients.

## Disclosures

The authors have no conflict of interest to declare.

This work was supported by NINDS at the NIH (R01 NS094384, S.A.H.; R01 NS103803, M.P.K.).

## References

[1] Boullon L Finn DP Llorente-Berzal Á. Sex differences in a rat model of peripheral neuropathic pain and associated levels of endogenous cannabinoid ligands. Front Pain Res 2021;2:1–12. 10.3389/fpain.2021.673638 PMC8915733 35295501

[2] Bourquin AF Süveges M Pertin M Gilliard N Sardy S Davison AC Spahn DR Decosterd I. Assessment and analysis of mechanical allodynia-like behavior induced by spared nerve injury (SNI) in the mouse. PAIN 2006;122:14.e1–14.e14. 16542774 10.1016/j.pain.2005.10.036

[3] Casals-Díaz L Vivó M Navarro X. Nociceptive responses and spinal plastic changes of afferent C-fibers in three neuropathic pain models induced by sciatic nerve injury in the rat. Exp Neurol 2009;217:84–95. 19416675 10.1016/j.expneurol.2009.01.014

[4] Collins ED Novak CB Mackinnon SE Weisenborn SA. Long-term follow-up evaluation of cold sensitivity following nerve injury. J Hand Surg Am 1996;21:1078–85. 8969435 10.1016/S0363-5023(96)80319-4

[5] Corbo J Zennou-Azogui Y Xerri C Catz N. Cortical merging in S1 as a substrate for tactile input grouping. eNeuro 2018;5:1–17. 10.1523/ENEURO.0342-17.2017 PMC5773279 29354679

[6] Darrow MJ Mian TM Torres M Haider Z Danaphongse T Rennaker RL Kilgard MP Hays SA. Restoration of somatosensory function by pairing vagus nerve stimulation with tactile rehabilitation. Ann Neurol 2020;87:194–205. 31875975 10.1002/ana.25664 PMC9624178

[7] Donoghue JP Suner S Sanes JN. Dynamic organization of primary motor cortex output to target muscles in adult rats II. Rapid reorganization following motor nerve lesions. Exp Brain Res 1990;79:492–503. 2340869 10.1007/BF00229319

[8] Duff SV. Impact of peripheral nerve injury on sensorimotor control. J Hand Ther 2005;18:277–91. 15891985 10.1197/j.jht.2005.02.007

[9] Dunnett SB Torres EM Annett LE. A lateralised grip strength test to evaluate unilateral nigrostriatal lesions in rats. Neurosci Lett 1998;246:1–4. 9622193 10.1016/s0304-3940(98)00194-3

[10] Galtrey CM Fawcett JW. Characterization of tests of functional recovery after median and ulnar nerve injury and repair in the rat forelimb. J Peripher Nerv Syst 2007;12:11–27. 17374098 10.1111/j.1529-8027.2007.00113.x

[11] Ganzer PD Darrow MJ Meyers EC Solorzano BR Ruiz AD Robertson NM Adcock KS James JT Jeong HS Becker AM Goldberg MP Pruitt DT Hays SA Kilgard MP Rennaker RL. Closed-loop neuromodulation restores network connectivity and motor control after spinal cord injury. Elife 2018;7:e32058. 29533186 10.7554/eLife.32058 PMC5849415

[12] Garraghty PE Kaas JH. Dynamic features of sensory and motor maps. Curr Opin Neurobiol 1992;2:522–7. 1525553 10.1016/0959-4388(92)90191-m

[13] Grinsell D Keating CP. Peripheral nerve reconstruction after injury: a review of clinical and experimental therapies. Biomed Res Int 2014;2014:1–13. 10.1155/2014/698256 PMC4167952 25276813

[14] Hewedy MA Abdelwahab OM. Surgical management of traumatic radial nerve injury. Egypt J Neurosurg 2016;31:195–200.

[15] Hulsey DR Mian TM Darrow MJ Hays SA. Quantitative assessment of cortical somatosensory digit representations after median and ulnar nerve injury in rats. Exp Brain Res 2019;237:2297–304. 31273391 10.1007/s00221-019-05593-0 PMC6679757

[16] Irwin MS Gilbert SEA Terenghi G Smith RW Green CJ. Cold intolerance following peripheral nerve injury. J Hand Surg Am 1997;22:308–16. 10.1016/s0266-7681(97)80392-0 9222907

[17] Jaggi AS Singh N. Role of different brain areas in peripheral nerve injury-induced neuropathic pain. Brain Res 2011;1381:187–201. 21238432 10.1016/j.brainres.2011.01.002

[18] Latef TJ Bilal M Vetter M Iwanaga J Oskouian RJ Tubbs RS. Injury of the radial nerve in the arm: a review. Cureus 2018;10:e2199. 29666777 10.7759/cureus.2199 PMC5902095

[19] Li R Hettinger PC Machol JA Liu X Stephenson JB Pawela CP Yan JG Matloub HS Hyde JS. Cortical plasticity induced by different degrees of peripheral nerve injuries: a rat functional magnetic resonance imaging study under 9.4 Tesla. J Brachial Plex Peripher Nerve Inj 2013;8:1–11. 23659705 10.1186/1749-7221-8-4 PMC3659007

[20] Lindsey AE Loverso RL Tovar CA Hill CE Beattie MS Bresnahan JC. An analysis of changes in sensory thresholds to mild tactile and cold stimuli after experimental spinal cord injury in the rat. Neurorehabil Neural Repair 2000;14:287–300. 11402879 10.1177/154596830001400405

[21] Ljungquist KL Martineau P Allan C. Radial nerve injuries. J Hand Surg Am 2015;40:166–72. 25442768 10.1016/j.jhsa.2014.05.010

[22] Lundborg G. Nerve injury and repair—a challenge to the plastic brain. J Peripher Nerv Syst 2003;8:209–26. 14641646 10.1111/j.1085-9489.2003.03027.x

[23] Merzenich MM Kaas JH Wall J Nelson RJ Sur M Felleman D. Topographic reorganization of somatosensory cortical areas 3b and 1 in adult monkeys following restricted deafferentation. Neuroscience 1983;8:33–55. 6835522 10.1016/0306-4522(83)90024-6

[24] Meyer-Rosberg K Kvarnström A Kinnman E Gordh T Nordfors LO Kristofferson A. Peripheral neuropathic pain—a multidimensional burden for patients. Eur J Pain 2001;5:379–89. 11743704 10.1053/eujp.2001.0259

[25] Meyers E Sindhurakar A Choi R Solorzano R Martinez T Sloan A Carmel J Kilgard MP Rennaker RL Hays S. The supination assessment task: an automated method for quantifying forelimb rotational function in rats. J Neurosci Methods 2016;266:11–20. 26976724 10.1016/j.jneumeth.2016.03.007 PMC5081185

[26] Meyers EC Granja R Solorzano BR Romero-Ortega M Kilgard MP Rennaker RL Hays S. Median and ulnar nerve injuries reduce volitional forelimb strength in rats. Muscle Nerve 2017;56:1149–54. 28120500 10.1002/mus.25590 PMC5589485

[27] Meyers EC Kasliwal N Solorzano BR Lai E Bendale G Berry A Ganzer PD Romero-Ortega M Rennaker RL Kilgard MP Hays SA. Enhancing plasticity in central networks improves motor and sensory recovery after nerve damage. Nat Commun 2019;10:5782. 31857587 10.1038/s41467-019-13695-0 PMC6923364

[28] Meyers EC Solorzano BR James J Ganzer PD Lai ES Rennaker RL Kilgard MP Hays SA. Vagus nerve stimulation enhances stable plasticity and generalization of stroke recovery. Stroke 2018;49:710–17. 29371435 10.1161/STROKEAHA.117.019202 PMC6454573

[29] Navarro X Vivó M Valero-Cabré A. Neural plasticity after peripheral nerve injury and regeneration. Prog Neurobiol 2007;82:163–201. 17643733 10.1016/j.pneurobio.2007.06.005

[30] Noble J Munro CA Prasad VSSV Midha R. Analysis of upper and lower extremity peripheral nerve injuries in a population of patients with multiple injuries. J Trauma 1998;45:116–22. 9680023 10.1097/00005373-199807000-00025

[31] Novak CB Mackinnon SE. Evaluation of cold sensitivity, pain, and quality of life after upper extremity nerve injury. Hand 2016;11:173–6. 27390558 10.1177/1558944715627633 PMC4920539

[32] O'Daly A Rohde C Brushart T. The topographic specificity of muscle reinnervation predicts function. Eur J Neurosci 2016;43:443–50. 26332647 10.1111/ejn.13058 PMC4738089

[33] Pruitt DT Danaphongse TT Lutchman M Patel N Reddy P Wang V Parashar A Rennaker RL Kilgard MP Hays SA. Optimizing dosing of vagus nerve stimulation for stroke recovery. Transl Stroke Res 2020;11:108–21. 32583333 10.1007/s12975-020-00829-6 PMC7759576

[34] Pruitt DT Danaphongse TT Schmid AN Morrison RA Kilgard MP Rennaker RL Hays SA. Traumatic brain injury occludes training-dependent cortical reorganization in the contralesional hemisphere. J Neurotrauma 2017;34:2495–503. 28462608 10.1089/neu.2016.4796 PMC5576212

[35] Rivera JC Glebus GP Cho MS. Disability following combat-sustained nerve injury of the upper limb. Bone Joint J 2014;96-B:254–8. 24493193 10.1302/0301-620X.96B2.31798

[36] Robinson LR. Traumatic injury to peripheral nerves. Muscle Nerve 2000;23:863–73. 10842261 10.1002/(sici)1097-4598(200006)23:6<863::aid-mus4>3.0.co;2-0

[37] Rosén B. Recovery of sensory and motor function after nerve repair. J Hand Ther 1996;9:315–27. 8994006 10.1016/s0894-1130(96)80037-8

[38] Sanes JN Suner S Lando JF Donoghue JP. Rapid reorganization of adult rat motor cortex somatic representation patterns after motor nerve injury. Proc Natl Acad Sci U S A 1988;85:2003–7. 3162322 10.1073/pnas.85.6.2003 PMC279910

[39] Schallert T Fleming SM Leasure JL Tillerson JL Bland ST. CNS plasticity and assessment of forelimb sensorimotor outcome in unilateral rat models of stroke, cortical ablation, parkinsonism and spinal cord injury. Neuropharmacology 2000;39:777–87. 10699444 10.1016/s0028-3908(00)00005-8

[40] Silva AC Rasey SK Wu X Wall JT. Initial cortical reactions to injury of the median and radial nerves to the hands of adult primates. J Comp Neurol 1996;366:700–16. 8833117 10.1002/(SICI)1096-9861(19960318)366:4<700::AID-CNE9>3.0.CO;2-8

[41] Taylor CA Braza D Rice JB Dillingham T. The incidence of peripheral nerve injury in extremity trauma. Am J Phys Med Rehabil 2008;87:381–5. 18334923 10.1097/PHM.0b013e31815e6370

[42] Vela F Martínez-Chacón G Ballestín A Campos J Sánchez-Margallo F Abellán E. Animal models used to study direct peripheral nerve repair: a systematic review. Neural Regen Res 2020;15:491–502. 31571661 10.4103/1673-5374.266068 PMC6921335

[43] Wall J Huerta M Kaas J. Changes in the cortical map of the hand following postnatal ulnar and radial nerve injury in monkeys: organization and modification of nerve dominance aggregates. J Neurosci 1992;12:3456–65. 1527590 10.1523/JNEUROSCI.12-09-03456.1992 PMC6575727

[44] Wall J Kaas J Sur M Nelson R Felleman D Merzenich M. Functional reorganization in somatosensory cortical areas 3b and 1 of adult monkeys after median nerve repair: possible relationships to sensory recovery in humans. J Neurosci 1986;6:218–33. 3944620 10.1523/JNEUROSCI.06-01-00218.1986 PMC6568627

[45] Woolf CJ Decosterd I. Spared nerve injury: an animal model of persistent peripheral neuropathic pain. PAIN 2000;87:149–58. 10924808 10.1016/S0304-3959(00)00276-1

[46] Yi H Kim MA Back SK Eun JS Na HS. A novel rat forelimb model of neuropathic pain produced by partial injury of the median and ulnar nerves. Eur J Pain 2011;15:459–66. 20965754 10.1016/j.ejpain.2010.09.014

